# Gut Feelings and Broken Hearts: A Case of Small Bowel Obstruction Causing Takotsubo Cardiomyopathy

**DOI:** 10.7759/cureus.25707

**Published:** 2022-06-07

**Authors:** Alexander Benyovszky, Lorin Berman, Noelle Provenzano, Bharghava Nelluri, Scott Fredd

**Affiliations:** 1 Internal Medicine, Einstein Medical Center Montgomery, East Norriton, USA; 2 Cardiology, Einstein Medical Center Montgomery, East Norriton, USA

**Keywords:** stress-induced cardiomyopathy, adult cardiology, apical cardiomyopathy, small-bowel obstruction, tako-tsubo cardiomyopathy (ttc)

## Abstract

Takotsubo cardiomyopathy (TCM), also known as apical ballooning syndrome or stress-induced cardiomyopathy, typically presents with features of the acute coronary syndrome. It is characterized by left ventricular apical akinesis and transient systolic dysfunction in the absence of obstructive coronary artery disease. Although its pathogenesis remains unclear, it is thought to be a catecholamine surge that is produced following intense physical or emotional stress. We present a case of TCM in a patient with small bowel obstruction (SBO), which is a rare trigger.

## Introduction

Takotsubo cardiomyopathy (TCM), also known as apical ballooning syndrome, is a form of stress-induced cardiomyopathy. It was first reported in Japan in 1990. It received its name due to the pathologically dilated left ventricle (LV), which resembles a Japanese "octopus-pot" trap. This LV dysfunction tends to be transient and occurs in the absence of obstructive coronary artery disease. TCM typically presents with symptoms that mimic acute coronary syndrome and is commonly associated with acute physical and emotional stress. This association with stress suggests that catecholamines play a role, whether that be by diffuse catecholamine-induced microvascular spasm resulting in myocardial stunning [1], or by direct catecholamine-associated myocardial toxicity [2]. Over 90% of TCM cases are observed in postmenopausal women [3]. The symptoms of TCM are most typically chest pain, dyspnea, and syncope, respectively. However, cases of asymptomatic TCM have also been documented [4]. Though the pathogenesis of TCM is not well understood, its occurrence in patients with small bowel obstruction (SBO) is extremely rare. Given its diverse presenting symptomatology, it is highly likely that TCM is underdiagnosed in patients experiencing high levels of physiologic or emotional stress. While the prognosis for TCM is overall good, complications include incomplete resolution of left ventricular ejection fraction (LVEF) and apical thrombus. Recurrence has been noted in up to 10% of patients. Mortality rates were higher in males, with an overall disease mortality rate of 4.2% [5]. We present a 65-year-old female who presented with a small bowel obstruction, leading to the development of TCM.

## Case presentation

**Figure 1:**
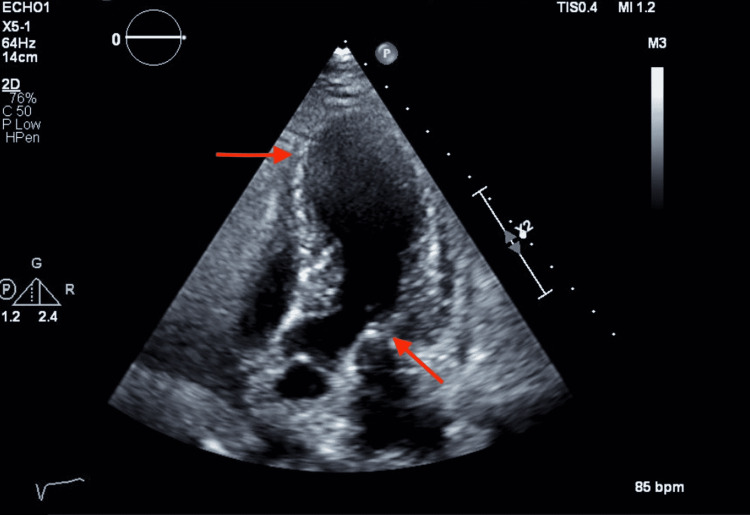
Echocardiography demonstrating the left ventricle with a hypokinetic apex with ballooning as denoted by the top arrow. Basal segments contract toward each other, as denoted by the bottom arrow. These forms the typical “octopus-pot” appearance.

A 65-year-old female with a history of chronic back pain following a motor vehicle accident, opioid and tobacco dependence, and endometriosis status post-open hysterectomy presented to the emergency department with a three-day history of right-sided abdominal pain and nausea. On initial presentation, she denied chest pain, shortness of breath, dizziness, or palpitations. She had no reports of fevers or any other gastrointestinal symptoms. Her EKG demonstrated T-wave inversions in leads V2-V6, with no ST elevations on the posterior EKG. Her initial blood work was significant for leukocytosis of 26.1 × 10^3^/mcL with neutrophilic predominance, hemoglobin of 15.5 gm/dL, lactic acid of 3.57 mmol/L, and a troponin of 2.06 ng/mL. A urine drug screen was positive for methamphetamine, cannabinoids, and opioids. Her physical examination was unremarkable and the patient denied any recent emotional triggers or recent stressors in her life. A computed tomography (CT) scan with contrast of the chest, abdomen, and pelvis did not show an aortic aneurysm or aortic dissection. However, there was air-filled distention of the small bowel without evidence of small bowel obstruction. Her initial echocardiogram showed a left ventricular ejection fraction of 25% with a large area of apical ballooning (Figure 1), consistent with TCM. 

The patient was started on metoprolol and was taken for an immediate cardiac catheterization. Findings from her catheterization revealed a patent left main coronary artery and a tortuous but patent right nondominant coronary artery. Post-procedure, she was transferred to the ICU and was started on dual antiplatelet therapy with aspirin and clopidogrel, atorvastatin, metoprolol, and ramipril. Her abdominal gradually worsened throughout the following days, and she developed acute hypotension. She was started on piperacillin-tazobactam for suspected intra-abdominal infection. Physical examination at that time demonstrated a distended and tympanic abdomen which was diffusely tender with high-pitched bowel sounds. An emergent CT angiography of the abdomen and pelvis was performed, which showed high-grade distal small bowel obstruction (SBO) due to volvulus in the distal small bowel mesentery with the most distal dilated loops appearing ischemic. A nasogastric tube was placed for decompression and the patient was subsequently taken for surgical exploration. She was found to have closed-loop small bowel obstruction at the level of the distal ileum with associated mesenteric torsion, which was surgically resected. A side-to-side functional end-to-end small bowel anastomosis was undertaken, and an abdominal wall fascial closure was performed. The patient's hospital course was further complicated by the development of a pelvic abscess, which required a pelvic drain to be placed. She was eventually discharged home following a 15-day hospital stay. Her gluteal drain was removed one day post-discharge. 

## Discussion

The clinical manifestations of TCM can range from asymptomatic or nonspecific symptoms to signs of heart failure, cardiogenic shock, and death. We report an uncommon case of a female patient with TCM accompanied by SBO. Although the initial presentation of our patient was not a clear SBO, it was consistent with an abdominal pathology. The usual TCM symptoms of chest discomfort were absent on admission. The diagnosis of TCM was confirmed by an elevated troponin level (2.06 ng/mL), an EKG showing T-wave inversions in leads V2-V6, with no ST elevation on posterior EKG, and subsequent echocardiography and cardiac catheterization, which demonstrated patent coronary vasculature, a decreased LVEF of 25%, and a large area of LV apical ballooning consistent with TCM (Figure 1). The catecholamine surge produced in response to stress is thought to be a trigger for TCM, though the precise mechanism is still unclear. These catecholamines potentially lead to cardiac endothelial dysfunction and myocardial injury. Upon literature review, we identified only three other cases of small bowel obstruction induced TCM. In 2018, Soliman et al. [6] reported two cases of takotsubo cardiomyopathy that developed after small bowel obstruction and were managed conservatively with bowel rest and pain control. In both cases, the patient’s subsequent echocardiography showed substantially improved ejection fractions. In 2014, Koci et al. [7] reported a case of TCM, which developed after necrotic small bowel obstruction and resolved completely after surgical management. Both Soliman et al. and Koci et al. also hypothesized that these intra-abdominal presentations of TCM might be secondary to catecholamine elevation due to their bowel obstruction. Though her presenting symptoms were intra-abdominal in nature, the discovery of elevated troponin and abnormal EKG prompted further investigation and led to the diagnosis of TCM before the SBO had fully manifested, making SBO the cause of TCM rather than its consequence. Further research is needed to determine the pathogenesis and exact mechanism of TCM. Though rare, there are cases of patients presenting with abdominal pain complicated by TCM [6-9]. Accurate and prompt diagnosis has substantial prognostic implications in view of the evidence that, in TCM patients, the underlying critical illness is the main driver of mortality rates [10]. Given this patient's nonspecific signs and symptoms, a high index of clinical suspicion is paramount for the prompt diagnosis of TCM. It is important to note that the patient's drug screen was positive on admission, which has also been linked to the development of TCM (although uncommon). Our case suggests that small bowel obstruction should be and accompanying catecholamine surge should be considered as a potential trigger leading to TCM.

## Conclusions

Takutsubo cardiomyopathy secondary to SBO is a rare presentation, seemingly the fourth clinical presentation of its kind. Identifying such cases helps in understanding the complex stress triggers associated with TCM. Although the exact pathogenesis of TCM is unclear, we know it is associated with acute physical and/or emotional stress. On this basis, TCM should be considered as part of a broader differential diagnosis in patients who present with intra-abdominal pathologies. For patients with an acute gastrointestinal disease requiring surgery, undiagnosed TCM and its concomitant decrease in LVEF can cause postoperative complications and poor outcomes. Further research is needed in order to determine the pathogenesis and exact mechanism of takotsubo cardiomyopathy.
